# Cystatin C and neutrophil gelatinase-associated lipocalin (NGAL) as novel biomarkers for monitoring acute kidney injury after cardiac surgery

**DOI:** 10.1186/1749-8090-8-S1-O259

**Published:** 2013-09-11

**Authors:** JM Kalisnik, A Hrastovec, M Skitek, A Jerin, B Gersak

**Affiliations:** 1 Department of Cardiovascular surgery, University Clinical Center Ljubljana, Slovenia; 2 Institute of Clinical Chemistry and Biochemistry, Ljubljana, Slovenia

## Background

Acute kidney injury (AKI) occurs frequently after cardiac surgery using cardiopulmonary bypass (CPB). Conventional markers of kidney function like creatinine and potassium concentration are unreliable indicators during acute changes in kidney function and demonstrate injury late in the clinical course. Therefore, in the search of more sensitive biomarkers for monitoring AKI following cardiac surgery we tested if cystatin C and neutrophil gelatinase-associated lipocalin (NGAL) measured after surgery would characterize AKI early after CPB.

## Methods

Fourty-four patients enrolled in the study met inclusion criteria. Serum and urinary NGAL, serum cystatin C and serum creatinine were determined on 5 time points pre-, peri- and postoperatively. Patients were divided into two groups- AKI group and non-AKI, depending on the increase in serum creatinine following surgery (based on AKIN (Acute Kidney Injury Network) classification AKI was defined as ≥ 50% or ≥ 0,3mg/dL rise in creatinine)). Results were compared between these two groups. P<0,05 was considered significant.

## Results

Nineteen patients (42%) developed postoperative AKI. The increase of serum cystatin C and serum creatinine in comparison to preoperative values was observed in majority of patients on the 1st day after surgery. Immediately after separating from CPB serum creatinine levels and serum cystatin C levels were lower than preoperative levels in both groups (AKI and non-AKI group). The difference between serum cystatin C levels on the 1st day after CPB between AKI and non-AKI group was considered significant (serum cystatin C: AKI: 1694±743 μg/L; non-AKI: 1048±302 μg/L; p=0,01; Independent-Sample Median test). The difference between urinary and serum NGAL levels after CPB between AKI and non-AKI group was not significant.

## Conclusions

An increase of post-CPB serum cystatin C is associated with AKI after cardiac surgery. Serum cystatin C has better potential to be used as a diagnostic biomarker for AKI following cardiac surgery as serum and urinary NGAL. The increase in serum cystatin C levels postoperatively occurred around the same time as increase in serum creatinine.

